# Early Diagnosis and Successful Treatment of Infectious Aortitis in a Hemodialysis Patient Through Physical Examination and Imaging

**DOI:** 10.7759/cureus.86112

**Published:** 2025-06-16

**Authors:** Rieko Higashide, Yoshitatsu Ohara, Tatsumi Kodama, Ryo Yasuhara, Takayuki Toda

**Affiliations:** 1 Department of Nephrology, Tsuchiura Kyodo General Hospital, Tsuchiura, JPN

**Keywords:** hemodialysis patient, infectious aortic aneurysms, pre-aneurysmal infectious, staphylococcus aureus, stent grafting

## Abstract

Infectious aortitis is rarely diagnosed before it becomes an infectious aortic aneurysm, and its optimal management remains less well established compared to that of infectious aortic aneurysms. We report a case of pre-aneurysmal infectious aortitis in a patient undergoing maintenance hemodialysis who presented with fever. Antibiotics were promptly initiated upon diagnosis, and the clinical course initially suggested good infection control. However, aneurysmal transformation eventually occurred, necessitating surgical intervention. This case highlights that, even in the absence of apparent clinical deterioration under antibiotics, aneurysm formation may still progress in cases of infectious aortitis. Therefore, careful and frequent imaging follow-up is essential for the early detection of aneurysmal changes.

## Introduction

Infectious aortitis is a condition characterized by infection of the aortic wall and is most often accompanied by aneurysm formation, in which case it is usually called an infected aortic aneurysm.

An infected aortic aneurysm is a serious condition that arises either from infection-induced aneurysmal degeneration of the aortic wall or secondary infection of a preexisting aneurysm. It carries a high risk of fatal complications such as rupture [1].

Once an aneurysm develops, the risk of fatal complications such as rupture increases significantly, and the condition is associated with high mortality. Therefore, prompt therapeutic intervention is essential. Standard management typically involves antibiotics for infection control, along with surgical intervention to prevent aneurysmal rupture [2].

Traditionally, anatomical bypass surgery was the primary method of surgical management; however, recent evidence suggests that endovascular treatment yields comparable outcomes. Due to its less invasive nature, endovascular intervention is increasingly becoming the preferred approach [3].

The most common causative organisms of infectious aortitis are *Staphylococcus* species, *Salmonella*, and *Escherichia coli* [4].

 Furthermore, infected aortic aneurysms caused by *Staphylococcus aureus* have been reported to be associated with lower survival rates compared to those caused by other organisms [5].

Among patients with infectious aortitis, some may present before the development of aneurysmal changes [6]. In such cases, the absence of aortic dilation makes diagnosis by imaging difficult, and rupture may occur even before aneurysm formation, making it a highly serious and potentially life-threatening condition [7].

Given the scarcity of reported cases, important aspects such as the differences in pathophysiology compared to infectious aortic aneurysms, the potential for conservative management with antibiotics alone, the risk of rupture, and the optimal timing for surgical intervention remain poorly understood.

Here, we report a case of pre-aneurysmal infectious aortitis. Although antibiotics were initiated promptly and the patient’s general and local inflammatory findings showed signs of improvement, aneurysm formation still occurred. Due to concern for rupture, semi-emergent endovascular treatment was performed. We present this case with a review of the literature, as it may provide new insights into the appropriate management of infectious aortitis diagnosed prior to aneurysm formation.

## Case presentation

An 81-year-old man undergoing maintenance hemodialysis for end-stage renal disease due to autosomal dominant polycystic kidney disease (ADPKD) presented with a fever of 38°C for eight days before admission. He visited our hospital the following day. Laboratory tests showed a mild inflammatory response, and chest and abdominal CT scans revealed no obvious infectious focus. His vital signs were stable, physical examination was unremarkable, and his overall condition appeared well; therefore, he was managed conservatively. Blood cultures taken at that time were negative.

Despite initial observation, the fever persisted at around 38°C. On the day of admission, repeat laboratory testing showed elevated inflammatory markers. Aside from fatigue associated with fever, the patient reported no other symptoms. At the time of re-evaluation, his blood pressure was 153/73 mmHg, pulse rate was 85 bpm, respiratory rate was 20 breaths per minute, SpO₂ was 98% on room air, and body temperature was 38.0°C. On physical examination, he had mild epigastric tenderness during inspiration and spinal extension.

Initial laboratory data are summarized in Table 1.

**Table 1:** Blood tests on admission

Test	Result on admission	Normal range
White blood count	9.4×10^9/L	3.6-11×10^9/L
Hemoglobin	126×10^9/L	130-180×10^9/L
Platelet count	158×10^9/L	140-400×10^9/L
TP	69 g/L	60-80 g/L
Albumin	32 g/L	41-51 g/L
Sodium	143 mmol/L	136-143 mmol/L
Potassium	3.2 mmol/L	3.5-5.0 mol/L
Creatinine	3.3 mg/dL	0.80-1.30 mg/dL
ALT	29 IU/L	13-61 IU/L
AST	14 IU/L	0-48 IU/L
γ-GTP	115 IU/L	0-50 IU/L
Total Bilirubin	0.8 mg/dL	0.2-1.2 mg/dL
CRP	27.09 mg/dL	0-0.3 mg/dL

**Figure 1:**
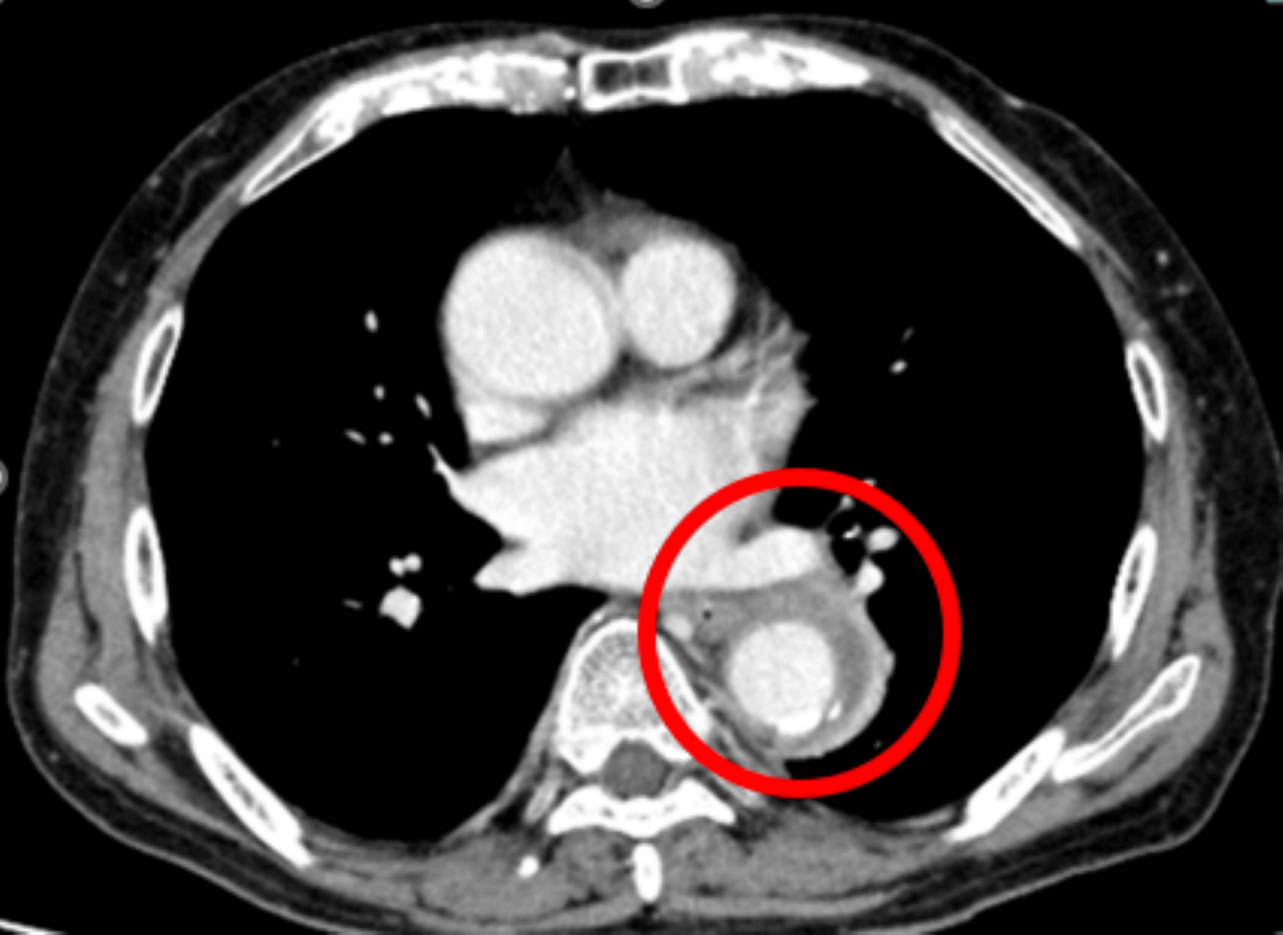
The horizontal slice of chest contrast enhanced CT on the day of admission The soft tissue density is seen around the descending thoracic aorta with mildly elevated adipose tissue density around the aorta (red round).

C-reactive protein (CRP) was markedly elevated at 27 mg/dL, while liver function tests, electrolytes, and blood counts were within normal limits. Transthoracic echocardiography revealed no abnormalities in cardiac function, wall motion, or evidence of vegetations. Contrast-enhanced CT of the neck, chest, and abdomen revealed a soft tissue density surrounding the descending thoracic aorta, with mild surrounding fat stranding (Figure 1). No abscess formation or embolic lesions were observed in other locations.

**Figure 2:**
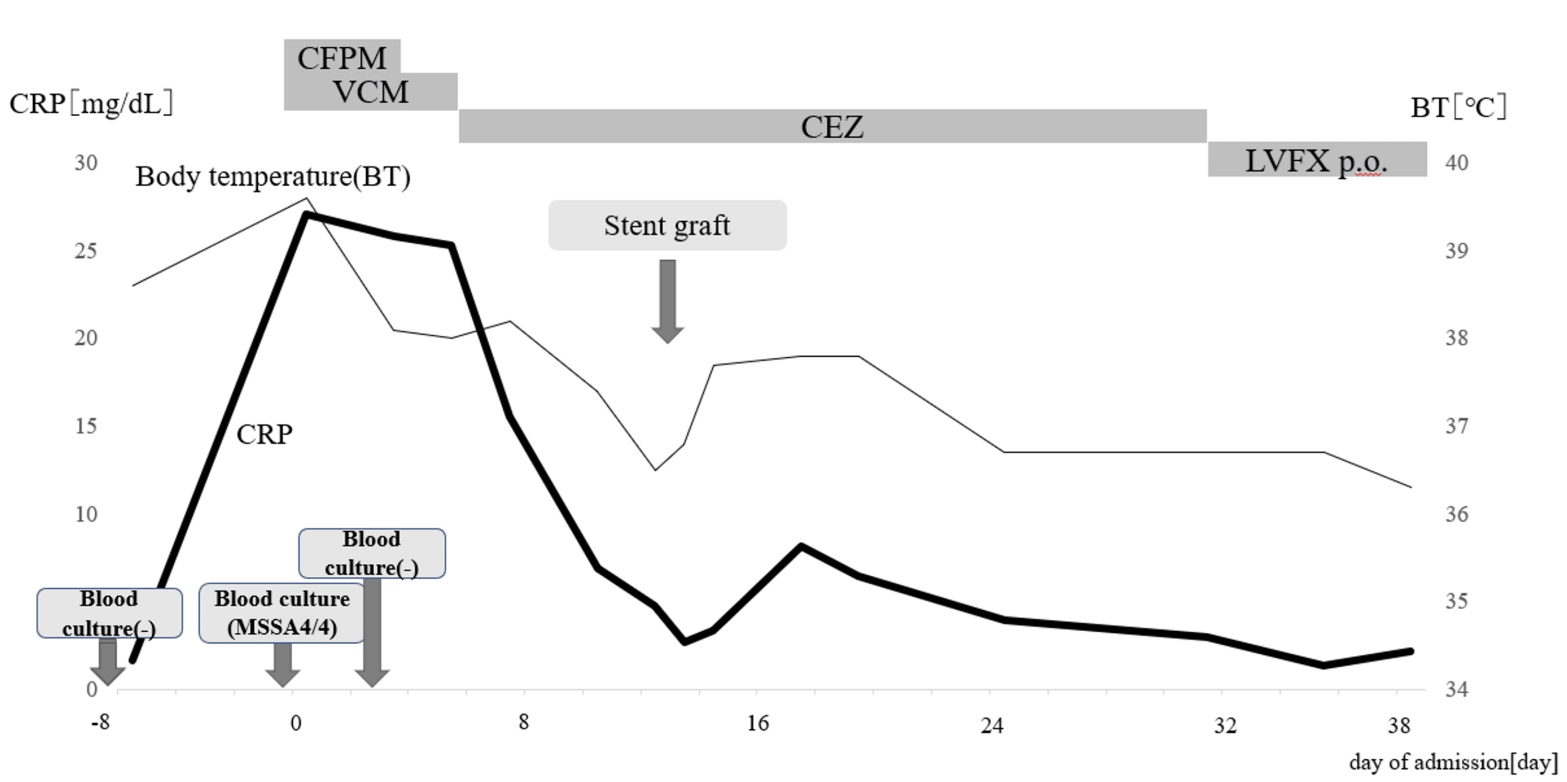
Clinical course

These findings were not present on imaging obtained on eight days before admission, indicating a rapid change within approximately one week. Given the persistent fever and physical examination findings, infectious aortitis was suspected as the primary diagnosis. The clinical course of the patient is illustrated in Figure 2.

Antibiotic therapy with cefepime (CFPM) and vancomycin (VCM) was initiated on the day of admission. On day 3 after admission, methicillin-sensitive *Staphylococcus aureus *(MSSA) was identified from blood cultures obtained at admission, prompting a switch to cefazolin (CEZ).

The patient became afebrile after starting antibiotics, and his CRP level began to decrease. Then, the previously noted epigastric tenderness during inspiration and spinal extension resolved.

**Figure 3:**
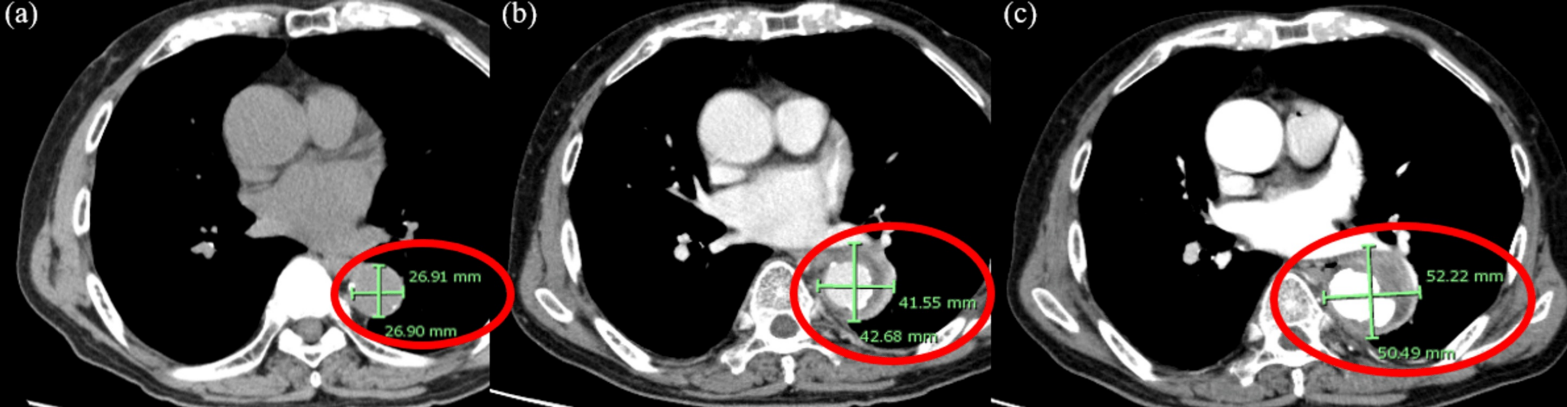
The horizontal slice of chest CT (a) The aortic diameter was 26.9 mm x 26.9 mm on eight days before admission(red round). It also showed calcification of the aortic wall. (b) The aortic diameter was 41.5 mm x 42.6 mm on the day of admission (red round). (c) The aortic diameter was 52.2 mm x 50.4 mm on day 6 after admission (red round), indicating an increase in aortic diameter with time.

However, a contrast-enhanced CT on day 6 after admission revealed an increase in aortic diameter and the formation of a protruding aneurysm (Figure 3).

At that point, emergency endovascular stent grafting was considered; however, due to persistently elevated inflammatory markers and ongoing fever, the infection was deemed to be uncontrolled. Following consultation with the vascular surgery team, a decision was made to continue conservative management with antibiotics while informing the patient of the potential risk of rupture. Subsequently, inflammatory markers began to decline, and blood cultures obtained on day 3 were confirmed negative by day 10. These findings suggested improved infection control, and since imaging performed on day 11 showed further aneurysmal enlargement, semi-emergent endovascular stent grafting was performed on day 13.

**Figure 4:**
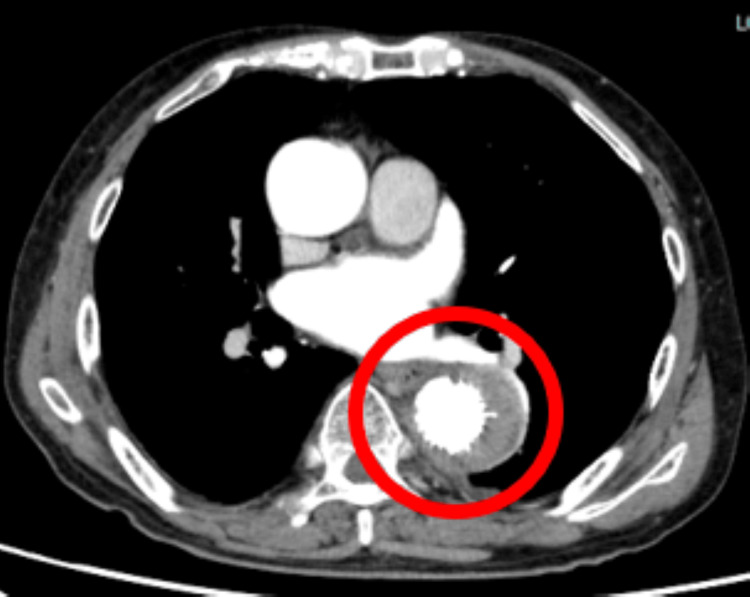
The horizontal slice of chest CT CT image after vascular stenting on 33 days after admission (red round). No signs of infection or endoleak are observed.

On CT imaging performed on day 18 after admission, there was no further aneurysmal enlargement, and inflammatory markers continued to improve. By day 33 after admission, the aneurysm showed signs of regression, and inflammation had resolved (Figure 4).

On day 34, intravenous antibiotics were switched to oral therapy. CEZ was suspected as a potential cause of a drug-induced rash that developed during the clinical course but resolved spontaneously. Taking this into consideration, and in accordance with the recommendation from the infectious diseases team, oral levofloxacin - a non-cephalosporin agent - was selected for long-term antibiotic therapy.

 As the patient remained afebrile and in good general condition, he was discharged on day 40 after admission with continued oral antibiotics.

## Discussion

The typical clinical features of infectious aortitis include fever (75%), chest or back pain (60%), abdominal pain (20%), and chills (16%) [6]. While pain, such as back or abdominal discomfort, is commonly observed, the symptoms are often nonspecific. Even in cases with aneurysmal transformation, fever and pain are reported in 84.1% and 75.0% of patients, respectively [8], yet the presentation remains largely nonspecific, making early diagnosis challenging. In the presented case, where fever is the only prominent symptom, diagnosis may be delayed until imaging is performed. Although this patient initially reported no specific symptoms during the interview, physical examination revealed mild pain in the epigastric region during deep inspiration and spinal extension.

In this case, the patient experienced epigastric pain during deep inspiration and spinal extension. In addition, contrast-enhanced CT revealed periaortic soft tissue attenuation - an imaging finding considered characteristic of infectious aortitis [9] - strongly suggesting an infectious focus in the descending aorta. Although these findings alone do not exclude other non-infectious causes of aortitis, such as inflammatory aortitis [2], they contributed to the early initiation of treatment under the presumption of potentially life-threatening infectious aortitis. Ultimately, aneurysmal transformation occurred, requiring invasive intervention with endovascular stent grafting. However, close monitoring with frequent imaging allowed surgical intervention to be performed before rupture, resulting in a successful clinical outcome.

Immunosuppression and atherosclerosis are considered key risk factors for infectious aortitis [10]. Patients on hemodialysis often present with both compromised immune function and advanced atherosclerosis, and hemodialysis itself can be a predisposing factor for infectious aortitis [11]. In this case, significant aortic calcification was noted (Figure 3). Hemodialysis patients are subject to frequent vascular access punctures, which can serve as a potential route of infection - *Staphylococcus aureus* is a common causative organism in such infections [12]. In our patient, MSSA was isolated from blood cultures, and no other evident source of infection was identified. Therefore, bacteremia resulting from vascular access puncture was considered the most likely cause of infection. Infectious aortitis should be considered in the differential diagnosis of prolonged fever in dialysis patients, even in the absence of pain.

The majority of infectious aortitis cases are associated with aneurysm formation, although it is often unclear whether the aneurysm is already present at the time of infection or develops as the infection progresses [2]. In our case, no aneurysm was observed at the time of diagnosis. Despite prompt initiation of antibiotics and subsequent improvement in symptoms and inflammatory markers, aneurysmal transformation still progressed; this is a noteworthy feature of the case. Once an aneurysm develops, the risk of rupture is approximately 60% [11], necessitating urgent intervention. In most cases, medical therapy alone is insufficient after aneurysmal formation, and surgical repair becomes essential. Conventional approaches include bypass surgery and graft replacement [2]; however, endovascular stent grafting has emerged as a viable alternative.

Endovascular aortic repair was first introduced for thoracic aortic aneurysms in 1998 [13] and has since been shown to yield outcomes comparable to open surgery, with the added benefit of lower invasiveness [3]. Given our patient’s advanced age and dialysis dependence, we opted for the less invasive endovascular approach. However, in the presence of uncontrolled infection, stent grafting carries risks such as stent graft infection, endoleak, migration, and eventual rupture [14]. For small, asymptomatic infectious aneurysms without signs of enlargement or perfusion compromise, prolonged intravenous antibiotics may be a viable conservative option [15].

In our case, no aneurysmal change was observed at the time of diagnosis. Since a larger aneurysm diameter is generally associated with a higher risk of rupture [16], the risk of rupture was considered to be low in this patient.

Aneurysm formation was observed on day 6 after admission. Reports have indicated that the mortality rate was lower in groups undergoing surgery after infection control compared to those operated on early before infection was controlled [17,18].

Therefore, we initially pursued conservative antibiotics to avoid postoperative infectious complications or recurrence.

Nevertheless, aneurysm formation progressed without accompanying symptoms, despite apparent clinical control of the infection. This suggests that even when systemic infection appears well-controlled, the risk of aneurysm progression remains. Consequently, as with infectious aortic aneurysms, the optimal strategy may be to initiate surgical repair as early as possible following diagnosis and antibiotics.

Reports exist of cases similar to ours in which antibiotics alone ultimately failed to prevent aneurysm formation, requiring surgical intervention [19]. Furthermore, in situations where surgery is delayed due to concerns about persistent or recurrent infection - as in our case - frequent imaging and meticulous follow-up are essential, even when local infection appears well controlled. There are also reports of rupture occurring prior to detectable aneurysmal changes [7,20], underscoring the importance of timely surgical decision-making.

## Conclusions

We report a case of infectious aortitis with fever as the sole symptom, without any associated pain or other obvious complaints. Despite a favorable clinical course with antibiotic therapy, aneurysmal transformation occurred, and ultimately, endovascular stent placement was required for intervention. Aortitis often presents with nonspecific symptoms and, in many cases, may lack overt clinical signs. While careful physical examination is crucial, the key challenge lies in the suspicion of the condition itself. In infectious aortitis, even if local infection appears well-controlled with antibiotics, aneurysmal formation and rupture remain possible. Therefore, frequent imaging is necessary, and timely surgical intervention should be considered based on these findings.
